# Two-year follow-up study (PRIMROSE 3) to assess bone mineral density in subjects with uterine fibroids completing the PRIMROSE 1 and PRIMROSE 2 linzagolix trials

**DOI:** 10.1093/hropen/hoaf025

**Published:** 2025-05-13

**Authors:** Jacques Donnez, Felice Petraglia, Hugh Taylor, Christian Becker, Sven Becker, Francisco Carmona Herrera, Maciej Paszkowski, Elke Bestel, Satoshi Hori, Marie-Madeleine Dolmans

**Affiliations:** Department of Gynecology, Université Catholique de Louvain, Brussels, Belgium; Department of Gynecology, Société de Recherche pour l’Infertilité (SRI), Brussels, Belgium; Obstetrics and Gynecology Unit, Department of Clinical Experimental and Biomedical Sciences, University of Florence, Florence, Italy; Department of Obstetrics, Gynecology and Reproductive sciences, Yale School of Medicine, New Haven, CT, USA; Nuffield Department of Women’s & Reproductive Health, Endometriosis CaRe Centre, University of Oxford, Oxford, UK; Department of Gynecology and Obstetrics, Frankfurt Goethe University, Frankfurt, Germany; Endometriosis Unit, Hospital Clinic of Barcelona, University of Barcelona, Barcelona, Spain; Department of Gynaecology, Medical University of Lublin, Lublin, Poland; Department of Medical Affairs, Theramex UK Ltd, London, UK; Department of Medical Affairs, Theramex UK Ltd, London, UK; Gynecology Research Laboratory, Department of Gynecology, Institut de Recherche Expérimentale et Clinique, Université Catholique de Louvain, Brussels, Belgium; Gynecology Department, Cliniques Universitaires St-Luc, Brussels, Belgium

**Keywords:** bone mineral density, BMD, GnRH antagonist, linzagolix, add-back therapy, ABT

## Abstract

**STUDY QUESTION:**

How important was the change in lumbar spine (L1–L4), femoral neck, and total hip bone mineral density (BMD) from post-treatment baseline values to 24 months after the end of treatment in PRIMROSE 1 and PRIMROSE 2 study participants?

**SUMMARY ANSWER:**

In the present study (PRIMROSE 3), mean percentage changes in lumbar spine BMD from the post-treatment baseline to month 24 (primary endpoint) were small in most treatment groups and similar to variations in the placebo group.

**WHAT IS KNOWN ALREADY:**

Due to its mechanism of action, some BMD decreases are observed with oral GnRH antagonist treatment, depending on the dose administered and addition or not of add-back therapy (ABT) (1 mg oestradiol and 0.5 mg norethisterone acetate). In PRIMROSE 1 and PRIMROSE 2, no significant changes in BMD were observed in any of the three anatomic sites investigated (lumbar spine, femoral neck, and total hip) in any of the treated groups but one. Indeed, at 24 weeks, mean differences were most pronounced in the lumbar spine in participants given 200 mg linzagolix alone.

**STUDY DESIGN, SIZE, DURATION:**

PRIMROSE 3 is a long-term follow-up study on BMD in subjects who completed at least 20 weeks of treatment in the main linzagolix trials (PRIMROSE 1 or PRIMROSE 2) and underwent dual-energy X-ray absorptiometry (DEXA) within 35 days of their last treatment (week 24 or week 52 [extension study]). The primary endpoint was the change in lumbar spine (L1–L4), femoral neck, and total hip BMD from post-treatment baseline values to 24 months after the end of treatment in PRIMROSE 1 and PRIMROSE 2 study participants. The secondary endpoint was the change in lumbar spine (L1–L4), femoral neck, and total hip BMD from pre-treatment baseline values to 24 months after the end of treatment. The study involved an eligibility visit and up to three follow-up consultations at 12, 18 and/or 24 months after the end of treatment in either PRIMROSE 1 or PRIMROSE 2.

**PARTICIPANTS/MATERIALS, SETTING, METHODS:**

Patients given an end-of-treatment DEXA scan within 35 days of their last treatment were invited to participate in the PRIMROSE 3 study. Those who were pregnant or unable to undergo a DEXA scan on the same type of equipment as used for the end-of-treatment DEXA scan in PRIMROSE 1 or PRIMROSE 2 were not eligible for this trial. A total of 137 subjects were screened, 134 (97.8%) of whom were enrolled and 130 (94.9%) included in the safety analysis set. Subject groups were small and ranged from 7 subjects (placebo group) to 30 subjects (200 mg/200 mg+ABT group). Most subjects (n = 110, 80.3%) completed the study by evaluation of their BMD by DEXA at 2 years post-treatment.

This study (EudraCT number: 2021-000452-19) was conducted at 3 sites in Bulgaria, 4 sites in the Czech Republic, 4 sites in Hungary, 1 site in Latvia, 6 sites in Poland, 1 site in Romania, 5 sites in Ukraine, and 32 sites in the USA.

**MAIN RESULTS AND THE ROLE OF CHANCE:**

The most notable percentage increase from the end of treatment to month 24 was in the 200 mg/200 mg+ABT treatment group, which was also the group showing the greatest mean percentage BMD loss during linzagolix treatment. This marked upturn in BMD after cessation of treatment demonstrated the crucial role of ABT.

Percentage changes in lumbar spine BMD from the pre-treatment baseline to month 24 (secondary endpoint) remained above −2% in all linzagolix treatment groups. Small BMD modifications observed from both the post-treatment and pre-treatment baseline to month 24 after the end of therapy may not have any clinically relevant impact on overall bone health of linzagolix-treated individuals, since the *Z*-score of most subjects was within the expected range for age. In addition, changes in BMD values and *Z*-scores in the linzagolix treatment groups were mostly within the same range as in the placebo group.

**LIMITATIONS, REASONS FOR CAUTION:**

The number of patients is relatively small. Since interpretation of results from the month-12 and month-18 visits is limited due to the small number of subjects in each treatment arm at corresponding time points, giving rise to high data variability, this manuscript focuses on the month-24 visit only.

**WIDER IMPLICATIONS OF THE FINDINGS:**

It can be assumed that the small BMD changes observed from both the post-treatment and pre-treatment baseline to month 24 after cessation of therapy may not have any clinically relevant impact on overall bone health of linzagolix-treated individuals.

Changes in BMD values and *Z*-scores in the linzagolix treatment groups were mostly within the same range as in the placebo group, indicating that there are no long-term consequences on BMD after the end of linzagolix treatment.

**STUDY FUNDING/COMPETING INTEREST(S):**

Funding for the PRIMROSE studies was provided by ObsEva (Geneva, Switzerland). Analysis of data was partially supported by ObsEva (Geneva, Switzerland), Theramex (London, UK), and Kissei (Japan). Grant 5/4/150/5 was awarded to M.-M.D. by the FNRS.

J.D. was a member of the scientific advisory board of ObsEva and Preglem until 2023 and reports consulting fees from ObsEva, Gedeon Richter, and Theramex. F.P. has received consulting fees and honoraria for lectures from Theramex. H.T. has received grants from Abbvie, reports consulting fees from ObsEva and Gedeon Richter, has a patent on endometriosis biomarkers owned by Yale University, and was a past president of American Society of Reproductive Medicine (ASRM). C.B. was a member of the independent data monitoring board for the PRIMROSE trials and member of the advisory board for Spirit 1 and 2 trials. He was also the Chair for the ESHRE endometriosis guideline committee. Consulting fees from Myovant and Theramex went to the University of Oxford. S.B. has received consulting fees and honoraria for lectures from Theramex. F.C.H. reports consulting fees and honoraria for lectures, presentations, or educational events from Theramex and Gedeon Richter and receiving honoraria for participation in a data safety monitoring board for Organon. M.P. was a principal investigator in the ObsEva-sponsored PRIMROSE 2 and 3 trials. E.B. and S.H. are employees of Theramex. M.-M.D. has received fees for lectures from Gedeon Richter and Theramex.

**TRIAL REGISTRATION NUMBER:**

EudraCT number: 2021-000452-19.

WHAT DOES THIS MEAN FOR PATIENTS? Bone mineral density (BMD) changes in postmenopausal women are well documented. They are related to the decrease in circulating oestrogens after the menopause. BMD modifications are also reported in premenopausal adult women treated with therapies associated with BMD loss (chronic corticosteroids, injectable progestogen‐only contraceptives, GnRH agonists and antagonists). The efficacy of the GnRH antagonist linzagolix was established, for reduction of uterine fibroid-related symptoms. No significant changes in BMD were observed in any of the three anatomic sites (lumbar spine, femoral neck, and total hip) in any of the treated groups but one. At 24 weeks, mean BMD differences were most pronounced in the lumbar spine in participants given 200 mg linzagolix alone, with a drop of approximately 4%. The present study revealed that BMD modifications from the pre-treatment baseline and post-treatment baseline to month 24 after the end of therapy were small in most treatment groups and do not have long-term consequences on overall bone health.

## Introduction

While many articles are available on bone mineral density (BMD) changes in postmenopausal women, there are far fewer reports on BMD modifications in premenopausal adult women treated with therapies associated with BMD loss (chronic corticosteroids, injectable progestogen‐only contraceptives, GnRH agonists and antagonists, among others) (Wilkin *et al.*, 2010; Cohen and Shane, 2013; Nam *et al.*, 2013; Binkley *et al.*, 2017; Dagan *et al.*, 2017; Langdahl, 2017). The risk of BMD loss related to GnRH agonist therapy has indeed limited its administration to 6 months when given without add-back therapy (ABT). As expected, considering its mechanism of action, some BMD decreases have been observed with GnRH antagonist treatment depending on the dose administered and addition or not of ABT (Schlaff *et al.*, 2020; Al-Hendy *et al.*, 2021; Dolmans *et al.*, 2021; Donnez *et al.*, 2022).

In PRIMROSE 1 and PRIMROSE 2, two parallel, randomized, double-blind, placebo-controlled, phase 3 trials, the efficacy of the GnRH antagonist linzagolix was established with or without ABT (1 mg oestradiol and 0.5 mg norethisterone acetate (NETA)) for reduction of uterine fibroid-related symptoms (Donnez *et al.*, 2022, 2025). No significant changes in BMD were observed in any of the three anatomic sites (lumbar spine, femoral neck, and total hip) in any of the treated groups but one. At 24 weeks, mean BMD differences were most pronounced in the lumbar spine in participants given 200 mg linzagolix alone, with a 3.3% and 4.1% drop in PRIMROSE 1 and PRIMROSE 2, respectively. At 52 weeks, mean percentage decreases from baseline in the lumbar spine were 2.1% and 3.1%, respectively, in patients who had received 200 mg linzagolix up to week 24 and then switched to 200 mg with ABT, while a 0.8% reduction was seen in the placebo group (Donnez *et al.*, 2022). In all other therapy groups, mean BMD percentage loss was not significant (range 0.0–2.4%). Consistent with the small magnitude of observed decreases in BMD density, median BMD *Z*-scores at week 52 were >0 in all treatment groups (Donnez *et al.*, 2022). *Z*-scores compare individual BMD values to average values for the same age and gender.

The next logical step therefore entailed gathering longer-term data on the drug’s effect on BMD. To do so, the PRIMROSE 3 study was designed to collect these data on BMD (up to 24 months after the end of therapy) in order to evaluate drug pharmacodynamics and recovery in subjects completing at least 20 weeks of treatment in PRIMROSE 1 or PRIMROSE 2.

## Materials and methods

### Ethical approval

This study was conducted in compliance with the trial protocol registered with ClinicalTrials.gov (NCT03070899 and NCT03070951). Study interventions were limited and no investigational drugs were administered. Planned study procedures were no riskier to subjects than those associated with assessments in general clinical practice.

### Study design, patient population, and patient flow

**Figure 1.**
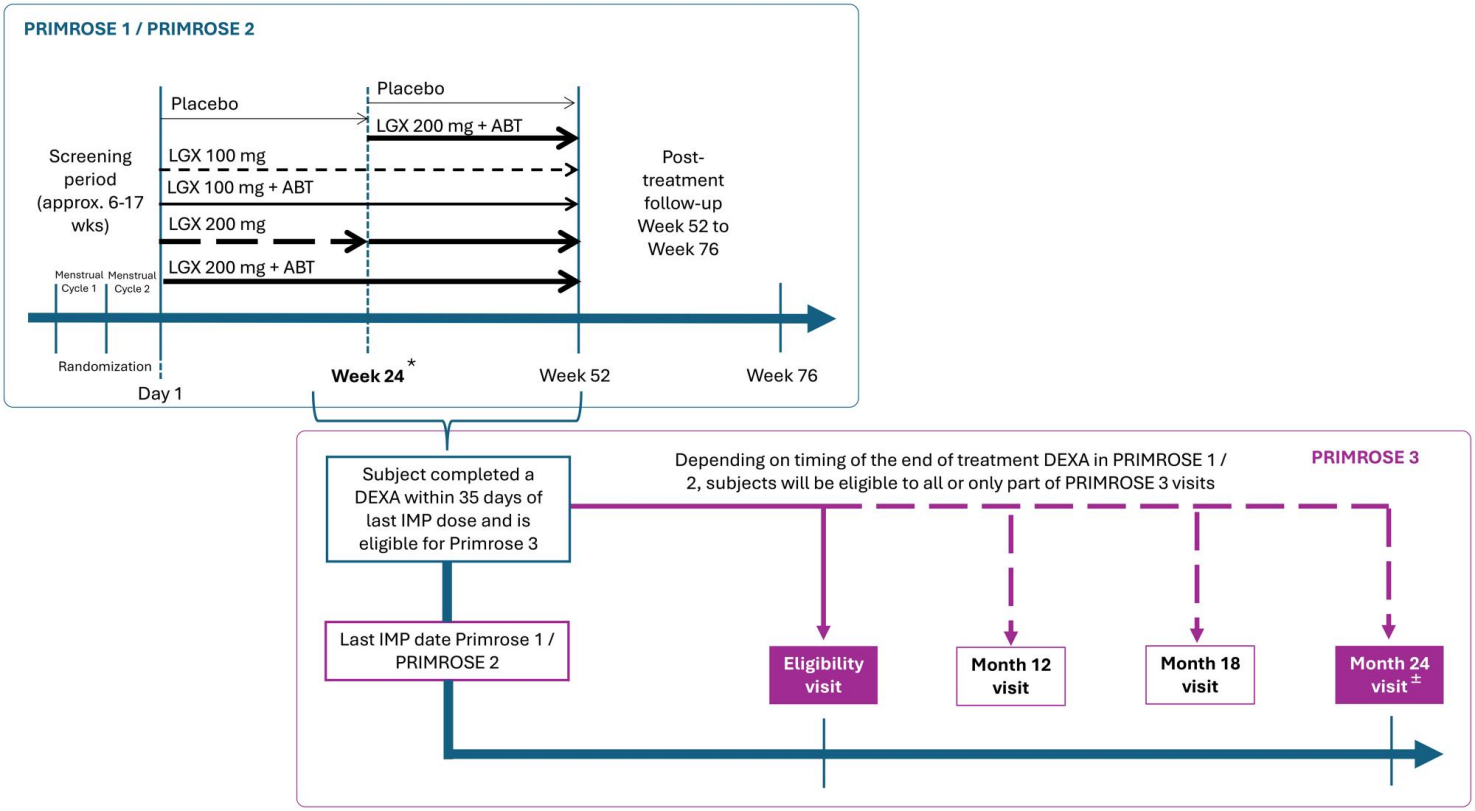
**Schematic of the study design.** ABT, add-back therapy (1 mg oestradiol/0.5 mg norethisterone acetate); DEXA, dual-energy X-ray absorptiometry; IMP, investigational medicinal product; LGX, linzagolix. *Women allocated to placebo or 200 mg Linzagolix alone were switched to 200 mg Linzagolix with ABT after 24 weeks, except in PRIMROSE 1, in which 50% of the women on placebo continued placebo (selected at random assignment) until week 52. ^±^Given the limited number of subjects in each treatment arm entering PRIMROSE 3, we focus on the reporting of month-24 visit only.

PRIMROSE 1 and PRIMROSE 2, two parallel, randomized, double-blind, placebo-controlled, phase 3 trials, were conducted with identical parameters over a treatment period of 52 weeks (Fig. 1). PRIMROSE 1 recruited women from 94 sites in the USA from May 2017 to October 2020. PRIMROSE 2 enrolled women from 95 sites in Europe as well as the USA from June 2017 to May 2020. Eligible subjects were women aged 18 years or older with ultrasonography-confirmed fibroids and heavy menstrual bleeding of at least 80 ml of menstrual blood loss per cycle.

PRIMROSE 1 started with 574 randomly assigned women, with 511 of them included in the full analysis set (FAS). PRIMROSE 2 randomized 535 women, 485 (91%) of whom were from Europe and 50 (9%) from the USA. Of these initial 535 randomly assigned subjects, 501 were included in the FAS. Eligible individuals were randomly allocated using an interactive web response system at a 1:1:1:1:1 ratio to be given one of the following daily regimens, administered orally: (i) placebo; (ii) 100 mg linzagolix only; (iii) 100 mg linzagolix plus hormonal ABT (1 mg oestradiol and 0.5 mg NETA); (iv) 200 mg linzagolix only; or (v) 200 mg linzagolix with hormonal ABT (1 mg oestradiol and 0.5 mg NETA). In the extension study, all women assigned to the placebo or 200 mg linzagolix alone groups in PRIMROSE 2 were switched to 200 mg linzagolix with ABT after 24 weeks. In PRIMROSE 1, 50% of subjects on a placebo continued with the placebo (random selection) until week 52. It is noteworthy that in PRIMROSE 1, 61–65% of women in the different arms were black vs 4–6% in PRIMROSE 2.

PRIMROSE 3 is a long-term follow-up study on BMD in subjects completing at least 20 weeks of treatment in the main linzagolix trials (PRIMROSE 1 or PRIMROSE 2) and undergoing dual-energy X-ray absorptiometry (DEXA) within 35 days of their last treatment (week 24 or week 52 [extension study]). The study involved an eligibility visit and up to three follow-up consultations at 12, 18 and/or 24 months after the end of treatment in either PRIMROSE 1 or PRIMROSE 2 (Fig. 1).

We were looking to include a large number of women eligible for evaluation by DEXA 24 months after completing linzagolix therapy. At the eligibility visit, subjects gave their informed consent before a comprehensive assessment of their eligibility criteria, collection of background data, and counselling on general bone health, including recommendations to take vitamin D and calcium supplementation. During all follow-up visits, subject data were collected on various aspects, such as medical conditions, concomitant medication, menopause status, and activity levels.

This study was conducted in compliance with the trial protocol and by trial personnel, who were fully qualified in terms of education, training, and experience. Study interventions were limited and no investigational drugs were administered. Planned study procedures, standard examinations, and questionnaires were no riskier to subjects than those associated with assessments in general clinical practice. Any adverse events related to trial procedures were documented. The previous trials were registered with ClinicalTrials.gov (NCT03070899 and NCT03070951). This study (EudraCT number: 2021-000452-19) was conducted at 3 sites in Bulgaria, 4 sites in the Czech Republik, 4 sites in Hungary, 1 site in Latvia, 6 sites in Poland, 1 site in Romania, 5 sites in Ukraine, and 32 sites in the USA.

Patients given an end-of-treatment DEXA scan within 35 days of their last treatment were invited to participate in the PRIMROSE 3 study. Those who were pregnant or unable to undergo a DEXA scan on the same type of equipment as used for the end-of-treatment DEXA scan in PRIMROSE 1 or PRIMROSE 2 were not eligible for this trial.

A total of 137 subjects were screened, 134 (97.8%) of whom were enrolled and 130 (94.9%) included in the safety analysis set, according to the previous regimen shown in Table 1. Subject groups were small and ranged from 7 (placebo group) to 30 (200 mg/200 mg+ABT group) individuals. Most subjects (n = 110, 80.3%) completed the study by evaluation of their BMD by DEXA at 2 years post-treatment. Of the 130 women in the safety set, 28 had a month-12 visit, 72 a month-18 visit, and 103 a month-24 visit. Since results from month-12 and month-18 were limited due to the small number of subjects in each treatment arm at corresponding time points, giving rise to high data variability, this manuscript focuses on the month-24 visit only. Moreover, the study was recruiting patients who might have experienced linzagolix-induced or age-related changes in BMD, so it was recommended that they take 1000 mg/day calcium and up to 600 IU vitamin D. Administration of vitamin D and calcium is thought to be beneficial for BMD recovery. Indeed, the National Institutes of Health and Institute of Medicine (IOM) advocate 1000 mg/day of elemental calcium and the IOM up to 600 IU/day of vitamin D year-round for postmenopausal women.

**Table 1.** Demographics and pre-treatment baseline characteristics (safety analysis set).

	Placebo	**Placebo**/**LGX 200 mg+ABT**	LGX 100 mg	LGX 100 mg+ABT	**LGX 200 mg**/**LGX 200 mg+ABT**	LGX 200 mg+ABT	Total
	(n = 7)	(n = 26)	(n = 22)	(n = 23)	(n = 30)	(n = 21)	(n = 130)
**Age (years)**							
Mean (SD)	39.9 (9.0)	42.2 (6.3)	43.9 (4.2)	43.0 (5.4)	41.6 (5.7)	44.7 (5.0)	42.8 (5.7)
Median	43.0	43.5	45.0	44.0	41.5	46.0	43.0
**Race, n (%)**							
Black	5 (71.4)	4 (15.4)	11 (50.0)	5 (21.7)	10 (33.3)	7 (33.3)	43 (33.1)
White	1 (14.3)	22 (84.6)	11 (50.0)	17 (73.9)	20 (66.7)	13 (61.9)	84 (64.6)
Other*	1 (14.3)	0	0	1 (4.3)	0	1 (4.8)	3 (2.4)
**Weight (kg)**							
Mean (SD)	80.37 (22.16)	73.89 (18.84)	92.32 (22.84)	82.32 (20.60)	86.00 (20.86)	88.11 (21.13)	83.94 (21.31)
Median	75.30	70.85	90.92	79.70	85.41	90.31	80.48
**BMI (kg**/**m^2^)**							
Mean (SD)	30.50 (9.25)	26.98 (7.01)	34.04 (6.67)	30.44 (7.64)	31.08 (7.01)	32.72 (8.24)	30.85 (7.60)
Median	29.86	25.98	34.78	30.08	30.45	32.78	29.75

ABT, add-back therapy (1 mg oestradiol/0.5 mg norethisterone acetate); LGX, linzagolix.

* Includes American Indian/Alaska Native, Native Hawaiian/Other Pacific Islander or Other.

Based on the above, the eligibility visit included counselling on general bone health, along with recommendations to take 1000 mg/day of calcium supplementation and up to 600 IU/day of vitamin D. Of the 130 patients in the study, 13 (10%) were on vitamin D, 9 (6.9%) on calcium, 5 (3.8%) on colecalciferol, 2 (1.5%) on calcium carbonate/colecalciferol, and 2 (1.5%) on calcium carbonate/vitamin D.

### Primary and secondary outcomes

The primary endpoint was the change in lumbar spine (L1–L4), femoral neck, and total hip BMD from post-treatment baseline values to 24 months after the end of treatment in PRIMROSE 1 and PRIMROSE 2 study participants. The secondary endpoint was the change in lumbar spine (L1–L4), femoral neck, and total hip BMD from pre-treatment baseline values to 24 months after the end of treatment.

### Data collection and acquisition

BMD was assessed in the femoral neck, total hip, and lumbar spine by DEXA at the end of the main study treatment period (namely week 24 or week 52) by a nominated trained primary technician at the DEXA facility. The same DEXA machine had to be used for all scans from an individual subject and they were all reviewed by a central imaging laboratory. There was also centralized monitoring of DEXA scan quality for each site, including a pre-qualification phantom scan and a monthly review of daily quality control (QC) data. If a scan did not meet QC standards, the site was asked to re-analyse or repeat the scan. Instructions on how to measure BMD and detailed information on centralized reading and QC were included in a specific imaging manual.

### Statistical analysis

A secondary analysis was conducted, based on primary findings from PRIMROSE 1 and 2. All subjects who completed at least 20 weeks of treatment in the main study and met the inclusion criteria were invited to participate in the PRIMROSE 3 study. It was estimated that up to 400 (39%) subjects from PRIMROSE 1 and PRIMROSE 2 would be eligible and willing to take part in PRIMROSE 3. However, several sites and subjects involved in PRIMROSE 1 and PRIMROSE 2 could not participate in the PRIMROSE 3 study, among other things due to the war in Ukraine, the COVID-19 pandemic, and difficulties recruiting patients for follow-up of 24 months. Only 134 out of the 400 anticipated subjects were therefore included in PRIMROSE 3.

No formal hypothesis testing was conducted. Primary and secondary endpoints were summarized by descriptive statistics for each time point, including summaries of change from baseline where applicable. Analyses were performed using the safety analysis set. Categorical analyses of BMD data (as far as applicable for individual endpoints) were conducted at each time point and included (i) continued bone loss if the subject was not completely recovered and BMD was less than the post-treatment baseline (% of subjects), (ii) partial recovery, if the subject was not completely recovered and BMD was greater than or equal to the post-treatment baseline (% of subjects), and (iii) complete recovery if BMD was greater than or equal to the pre-treatment baseline (% of subjects). All analyses were performed using the safety analysis set. Additional summaries were provided by subgroup analysis sets. Data from this study were held and evaluated by Cytel (Boston, MA, USA). All descriptive statistical analysis was conducted using SAS statistical software (version 9.4 or later) (SAS Institute, Cary, NC, USA).

## Results

### Demographic data

The majority of patients in the linzagolix treatment groups were white, ranging from 50.0% of subjects in the 100 mg without ABT group to 84.6% in the placebo/200 mg+ABT group. In the placebo group, the vast majority of subjects were black/African Americans (71.4%) (Table 1).

At pre-treatment baseline, mean (SD) age in the total population was 42.8 (5.7) years (min/max: 22/54 years) and mean (SD) BMI was 30.85 (7.60) kg/m^2^ (min/max: 18.4/56.0). Mean (SD) overall treatment duration was 50.95 (3.89) weeks, similar in all treatment groups. At the eligibility visit, most subjects in the treatment groups were premenopausal, ranging from 69.2% in the placebo/200 mg+ABT group to 87.0% in the 100 mg+ABT group.

### Bone mineral density

#### BMD changes from post-treatment baseline to 24 months after cessation of therapy

**Figure 2.**
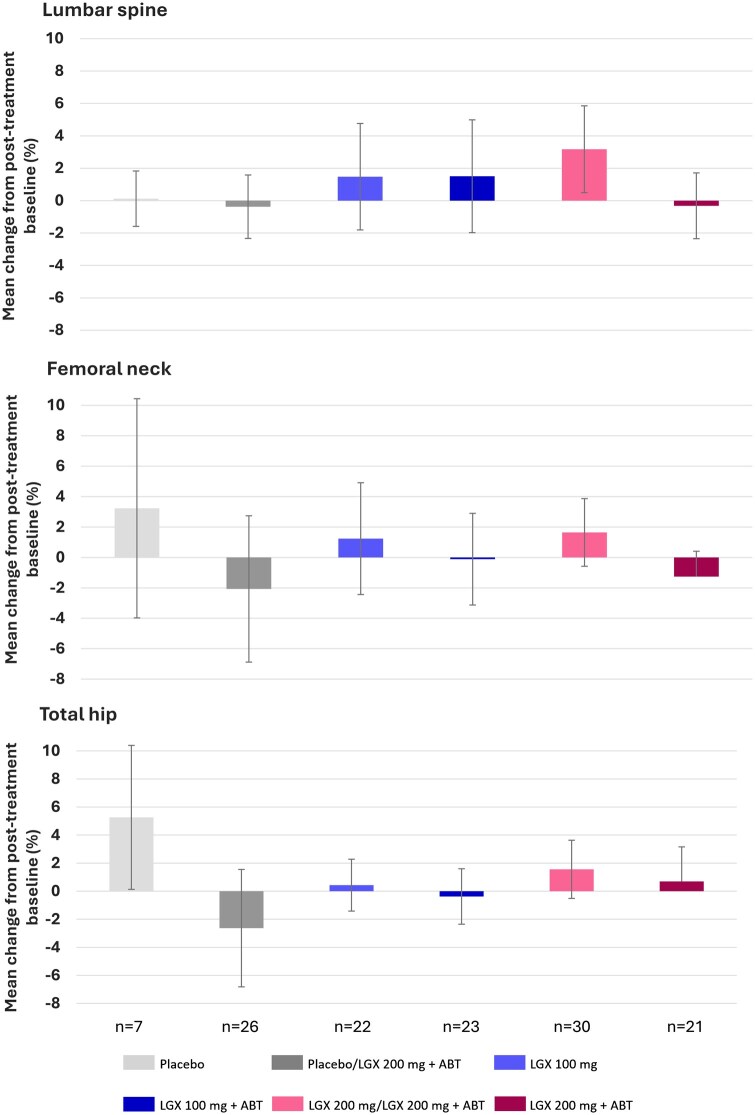
**Mean percentage changes from post-treatment baseline to visit month 24 in BMD by DEXA (safety analysis set).** ABT, add-back therapy (1 mg oestradiol/0.5 mg norethisterone acetate); BMD, bone mineral density; DEXA, dual-energy X-ray absorptiometry; LGX, linzagolix. Error bars denote 95% CIs.

In the PRIMROSE 3 safety population, end-of-treatment (namely post-treatment baseline) lumbar spine mean (SD) BMD values ranged between 1.044 (0.136) g/cm^2^ (placebo/200 mg+ABT) and 1.149 (0.132) g/cm^2^ (placebo). At the month-24 visit, mean (SD) percentage BMD changes from this post-treatment baseline varied from −0.375 (4.187) % in the placebo/200 mg+ABT group (95% CI: −2.334/1.584) to 3.173 (5.885) % in the 200 mg/200 mg+ABT group (95% CI: 0.494/5.852) (Fig. 2). Mean (SD) percentage changes from post-treatment baseline in the placebo group were minimal (0.120 [1.848] %, 95% CI: −1.589/1.830) and comparable to those in the placebo/200 mg+ABT (−0.375 [4.187] %, 95% CI: −2.334/1.584) and 200 mg+ABT (−0.319 [4.083], 95%CI: −2.349/1.712) groups.

In the femoral neck, mean (SD) BMD values at post-treatment baseline were between 0.839 (0.171) g/cm^2^ (placebo/200 mg+ABT) and 0.946 (0.112) g/cm^2^ (placebo). At the month-24 visit, mean (SD) percentage BMD changes from this post-treatment baseline ranged from −2.071 (10.565) % in the placebo/200 mg+ABT group (95% CI: −6.880/2.739) to 1.643 (4.749) % in the 200 mg/200 mg+ABT group (95% CI: −0.580/3.865). In the placebo group, the mean (SD) percentage change was 3.230 (7.789) % (95% CI: −3.974/10.434). No clear tendencies were observed in any of the treatment groups, as evidenced by the 95% CIs of mean percentage change.

In the total hip, mean (SD) BMD values at post-treatment baseline ranged from 0.896 (0.173) g/cm^2^ (placebo/200 mg+ABT) to 1.034 (0.142) g/cm^2^ (placebo). Mean (SD) percentage BMD changes from post-treatment baseline to the month-24 visit in the linzagolix treatment groups varied between −2.633 (9.196) % in the placebo/200 mg+ABT group (95% CI: −6.819/1.553) and 1.559 (4.431) % in the 200 mg/200 mg+ABT group (95% CI: −0.515/3.633). Mean (SD) percentage change in the placebo group was 5.263 (5.548) % (95% CI: 0.132/10.393).

An upturn in mean absolute change in *Z*-scores from post-treatment baseline to the month-24 visit was reported for most treatment groups at all assessment sites, ranging from 0.09 (lumbar spine in the 200 mg+ABT group) to 0.44 (lumbar spine 200 mg/200 mg+ABT group) in all linzagolix groups to 0.47 for the placebo group (total hip). No change or even slight downturn in mean absolute *Z*-scores was found in the placebo/200 mg+ABT group (femoral neck: 0.00, and total hip: −0.02) or the 200 mg+ABT group (femoral neck: −0.03).

#### BMD changes from pre-treatment baseline to 24 months after cessation of therapy

**Figure 3.**
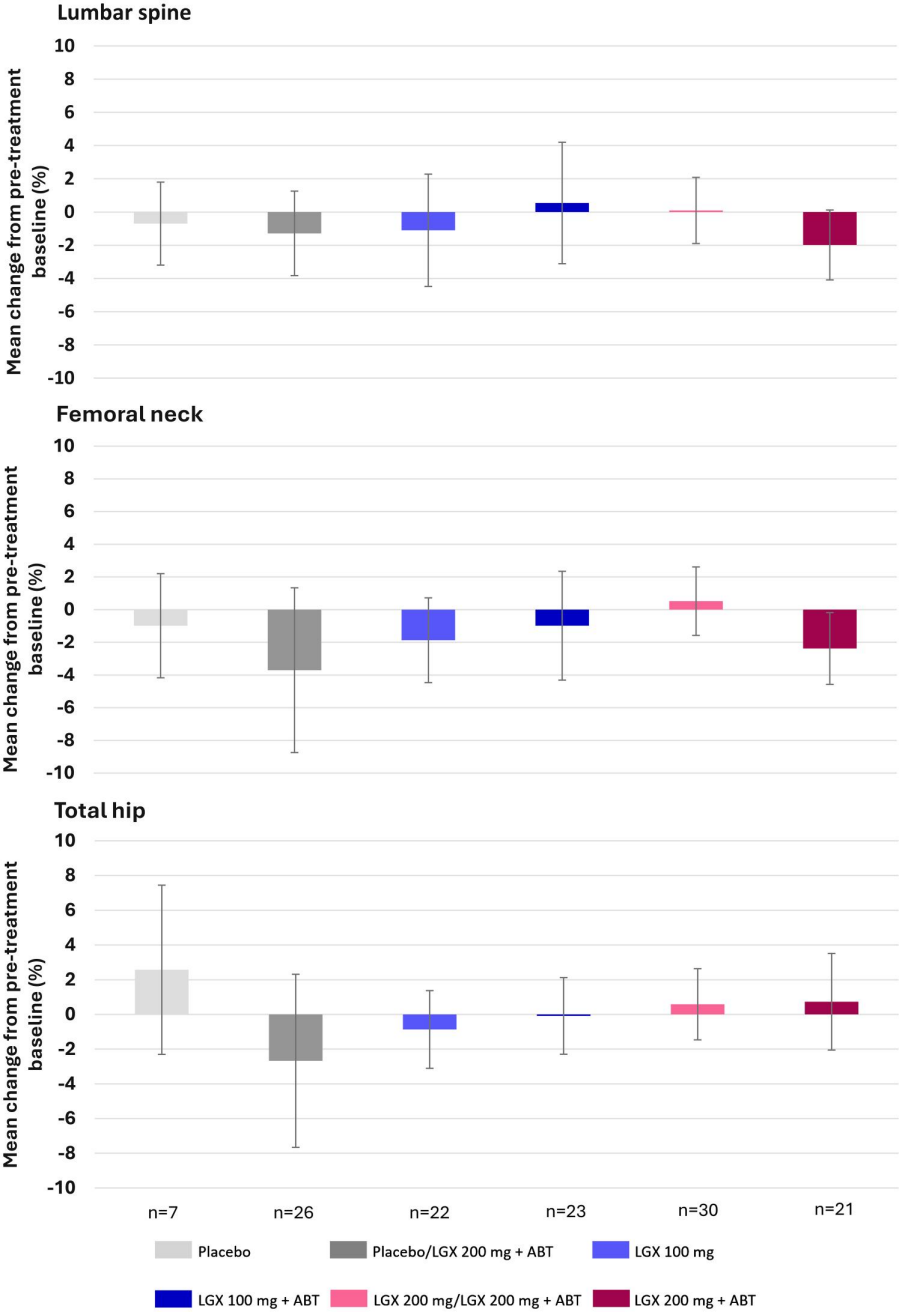
**Mean percentage changes from pre-treatment baseline to visit month 24 in BMD by DEXA (safety analysis set).** ABT, add-back therapy (1 mg oestradiol/0.5 mg norethisterone acetate); BMD, bone mineral density; DEXA, dual-energy X-ray absorptiometry; LGX, linzagolix. Error bars denote 95% CIs.

The secondary endpoint (Fig. 3) explored the change from pre-treatment baseline values in lumbar spine, femoral neck, and total hip BMD at 24 months after cessation of therapy.

**Figure 4.**
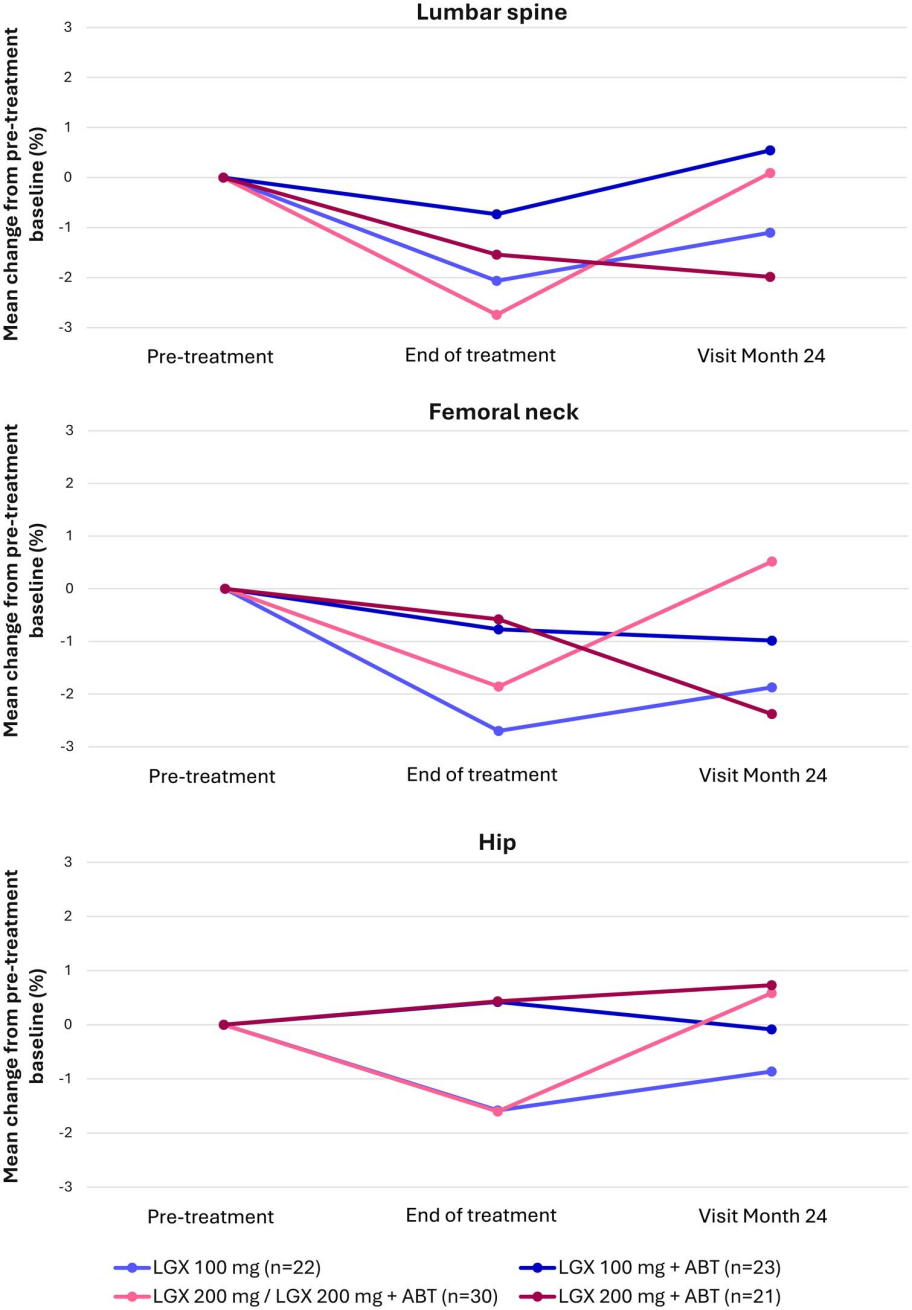
**Mean percentage changes in BMD of the lumbar spine by DEXA from pre-treatment baseline to end of treatment and visit month 24 (safety analysis set).** ABT, add-back therapy (1 mg oestradiol/0.5 mg norethisterone acetate); BMD, bone mineral density; DEXA, dual-energy X-ray absorptiometry; LGX, linzagolix.

In the PRIMROSE 3 safety population, end-of-treatment mean percentage changes in BMD (pre-treatment baseline) in lumbar spine had been most prominent in the 100 mg alone (−2.065 [3.948] %), 200 mg/200 mg+ABT (−2.742 [3.531] %) and 200 mg+ABT (−1.539 [3.129] %) groups. At 24 months post-treatment, mean percentage changes in lumbar spine BMD from pre-treatment baseline did not exceed −2% in any treatment group (Fig. 4). The greatest drop from pre-treatment baseline to month-24 post-treatment was encountered in the 200 mg+ABT group, with a mean (SD) percentage reduction of −1.985 (4.234) % (95% CI: −4.090/0.121).

For the femoral neck, greater BMD loss was reported in the placebo/200 mg+ABT group and the 200 mg+ABT group, with −3.706 (10.769) % and −2.378 (4.415) % of mean (SD) percentage loss. The upper limit of the 95% CI also remained below zero in the 200 mg+ABT group. For total hip values, mean (SD) percentage loss of BMD exceeding 2% was only reported in the placebo/200 mg+ABT group with −2.673 (10.657) %.

Mean absolute changes in *Z*-scores from baseline ranged from −0.09 (femoral neck in the placebo/200 mg+ABT group) to 0.19 (lumbar spine in the 200 mg/200 mg+ABT group and total hip in the 100 mg+ABT group) at the month-24 visit. In most treatment groups, *Z*-scores at 24 months were similar to pre-treatment baseline values.

#### Recovery rates

In most treatment groups, BMD status in the lumbar spine, femoral neck, and total hip was considered fully or partially recovered in at least 50% of subjects at 24 months after treatment cessation. Fewer than 50% of subjects who had at least partially recovered were only observed for femoral neck in the 200 mg+ABT group (38.9% subjects: 22.2% partially recovered, and 16.7% completely recovered), and for total hip in the placebo/200 mg+ABT group (45.0% subjects: all completely recovered) and the 100 mg+ABT group (47.1% subjects: 11.8% partially recovered and 35.3% completely recovered).

#### Analysis of the premenopausal subgroup

Overall, 77% of the safety population was premenopausal upon inclusion into PRIMROSE 3, the percentage ranging from 69.2% of subjects (n = 18) in the placebo/200 mg+ABT group to 87.0% (n = 20) in the 100 mg+ABT group. Analysis of the premenopausal subgroup revealed a marked mean (SD) percentage increase in BMD from post-treatment baseline to the month-24 visit in all three anatomic locations assessed in the 200 mg/200 mg+ABT group (lumbar spine: 4.461 [5.801] %, femoral neck: 2.574 [4.120] % total hip: 2.389 [4.048] %). Mean percentage changes in BMD from pre-treatment baseline to 24 months were below or around 1% in most treatment groups and generally comparable to changes in the placebo group. BMD status was considered partially or completely recovered in at least 50% of subjects 24 months after treatment cessation in all treatment groups, except the 200 mg+ABT group in the femoral neck (38.5%) and the 100 mg+ABT group in the total hip (40.0%). In all treatment groups and anatomic locations assessed, the proportion of subjects considered fully recovered was higher than the proportion of those deemed partially recovered.

## Discussion

This phase 3, long-term follow-up study (up to 24 months after the end of treatment) was designed to assess BMD changes in order to evaluate their dynamics and the recovery of bone loss in subjects with uterine fibroids who participated in PRIMROSE 1 or PRIMROSE 2 studies. BMD measurement by DEXA is currently the best way of identifying subjects at risk of osteoporotic fractures and should be performed in subjects with any risk factors (Beck *et al.*, 2021; FRAX^®^ Fracture Risk Assessment Tool, 2024). Indeed, the lower the BMD, the higher the risk of fracture. In women prior to menopause, a *Z*-score of −2.0 or lower is defined as ‘below the expected range for age’, while a *Z*-score above −2.0 is ‘within the expected range for age’. BMD is known to depend on race and BMI. African Americans are associated with higher BMDs than other racial groups in the USA (Wilkin *et al.*, 2010; Nam *et al.*, 2013; Binkley *et al.*, 2017). A higher BMI is also linked to elevated BMD at baseline, a typical subject with a BMI of 30 kg/m^2^ (class I, obese) estimated to show 5% higher baseline BMD values compared to a typical subject with a BMI of 18.5 kg/m^2^.

The risk of bone fractures due to reduced BMD in premenopausal women is low (Beck *et al.*, 2021). BMD changes should be considered in the wider context of BMD impacts associated with many other chronic medications (Chotiyarnwong and McCloskey, 2020). They are also associated with physiological events such as pregnancy and lactation, with up to 5% of BMD loss occurring in the lumbar spine (Winter *et al.*, 2020; Watts *et al.*, 2021).

While many articles have been published on BMD changes associated with fracture risk in postmenopausal women, relatively few describe changes in BMD in premenopausal adult women treated with drugs linked to BMD loss (chronic corticosteroids, injectable progestogen‐only contraceptives, GnRH agonists and antagonists) (Binkley *et al.*, 2017). One of the first articles (Devogelaer *et al.*, 1987) on BMD loss after GnRH agonist therapy was published back in 1987. Since then, a number of papers have documented the risk of BMD loss associated with GnRH agonist therapy-related hypo-oestrogenism (Wilkin *et al.*, 2010; Cohen and Shane, 2013; Nam *et al.*, 2013; Binkley *et al.*, 2017; Dagan *et al.*, 2017; Langdahl, 2017; FRAX^®^ Fracture Risk Assessment Tool, 2024), which is why its administration without ABT is limited to 6 months.

In the present article, we discuss BMD changes in relation to the administration of GnRH antagonists, and particularly linzagolix. We therefore first examined BMD changes reported in various studies on GnRH antagonists in the literature.

An extension study (6 months) (Simon *et al.*, 2020; Beck *et al.*, 2022) of Elaris UF-1 and UF-2 trials, evaluating the efficacy of elagolix, an oral GnRH antagonist, in the management of uterine fibroids-related symptoms revealed the mean lumbar spine BMD percentage change from baseline to be −4.8% in the elagolix alone (300 mg twice daily) treatment group, while the same dose with ABT (1 mg oestradiol and 0.5 mg NETA) yielded −1.5% after one year of treatment. In the same anatomical location after 6 months of treatment, the mean BMD percentage change from baseline was −2.8% with elagolix alone and −0.6% with elagolix plus ABT. Twelve months after therapy, it was −2.0% and −0.6%, respectively, demonstrating some degree of recovery. Similar trends were seen in total hip and femoral neck locations.

According to Osuga *et al.* (2021), mean changes in BMD from baseline to week 24 were −0.2% with the placebo and −1.6%, −2.6%, and −4.9% with 10, 20, and 40 mg relugolix, respectively, showing that BMD losses are greater at high doses of relugolix and related to the level and severity of induced hypo-oestrogenism. BMD changes were much less pronounced and below 1% when relugolix was administered with ABT for 24 weeks (1 mg oestradiol and 0.5 mg NETA under the name of relugolix-CT) (Al-Hendy *et al.*, 2021). Long-term (extension) studies confirmed the absence of significant BMD changes when relugolix was administered with ABT (Al-Hendy *et al.*, 2023).

In a dose-finding study into linzagolix, mean BMD (spine) loss at 24 weeks was <1% at doses of 50 and 75 mg, and increased in a dose-dependent manner up to 2.6% with 200 mg. BMD in the femoral neck and total hip showed a similar pattern (Donnez *et al.*, 2020). In the PRIMROSE 1 and PRIMROSE 2 trials, subjects were given 100 mg or 200 mg linzagolix (with or without ABT) or a placebo. At 24 weeks, mean differences were most prominent in the lumbar spine, showing BMD decreases of 3.3% and 4.1% in PRIMROSE 1 and PRIMROSE 2 respectively in participants given 200 mg linzagolix alone, compared to an increase of 0.4% and 0.5% in the placebo group (Donnez *et al.*, 2022). Following addition of ABT however, the percentage change from baseline to week 52 compared to week 24 improved, indicating a positive effect of ABT on BMD (Donnez *et al.*, 2022).

When ABT was co-administered with 100 or 200 mg linzagolix, mean (SD) BMD changes from baseline were for Primrose 1 respectively −0.914 (3.057) % and −0.839 (2.830) % at 24 weeks and 0.049 (2.101) % and −0.929 (3.008) % at 52 weeks in PRIMROSE 1, and −0.998 (2.435) % and −1.350 (2.575) % at 24 weeks and −1.479 (1.967) % and −2.026 (3.028) % at 52 weeks in PRIMROSE 2. From week 24 to week 52, rates of BMD change were less than −1% in the lumbar spine, suggesting plateauing of BMD loss (Donnez *et al.*, 2022).

The next step was to evaluate possible long-term effects on BMD and the degree of recovery in subjects involved in both PRIMROSE trials by collecting long-term data (24 months after the end of therapy). This was the goal of PRIMROSE 3. Of the 1037 subjects initially recruited in the PRIMROSE 1 and 2 studies, 130 subjects were included in the safety analysis set of PRIMROSE 3. Of those, 80% had undergone BMD evaluation by DEXA by month 24 after the end of therapy. The mean treatment duration of their linzagolix therapy in PRIMROSE 1 or 2 was 50.95 weeks (min/max: 23.9/53.1) and was similar between treatment groups. Treatment groups were also similar in terms of risk factors for BMD loss and attributable fractions during the PRIMROSE 3 study.

Changes in BMD were analysed in three specific anatomic sites: lumbar spine, femoral neck, and total hip. In the PRIMROSE 3 study, mean percentage changes in lumbar spine BMD from the post-treatment baseline (primary endpoint) to month 24 were small in most treatment groups and similar to changes in the placebo group. The most notable percentage increase from the end of treatment to month 24 was in the 200 mg/200 mg+ABT treatment group, which was also the group showing the greatest mean percentage BMD loss during linzagolix treatment. This marked upturn in BMD after cessation of therapy was also reflected in the highest absolute increase in mean score of all treatment groups, demonstrating the crucial role of ABT. For BMD in the femoral neck and total hip, no clear tendencies were spotted in mean percentage BMD change from post-treatment baseline to month 24 in any treatment group.

Percentage changes in lumbar spine BMD from the pre-treatment baseline to the month 24 (secondary endpoint) visit remained above −2% in all linzagolix treatment groups, the greatest decrease being −1.985**%** in the 200 mg+ABT group. Consistent with small mean percentage changes in BMD, mean absolute changes in *Z*-scores from pre-treatment baseline values to month 24 were also modest, ranging from −0.06 to 0.19 in the lumbar spine.

In the placebo group, mean percentage BMD and mean absolute changes seen in *Z*-scores were similar to values found in the linzagolix treatment groups. These findings suggest that the observed BMD changes with linzagolix result from age-related BMD decreases, since a similar reduction was actually encountered in an untreated population of comparable age. *Z*-scores of most subjects were also within the expected range for age.

In line with the small mean percentage BMD and mean absolute *Z*-score changes seen from pre-treatment baseline levels to month 24, more than half of the subjects overall achieved at least a partially recovered BMD status. Indeed, across the linzagolix treatment groups, proportions of subjects with partially or completely recovered BMD 24 months after treatment cessation ranged from 50.0% to 80.0%, similar to the placebo group, where the percentage of women with a BMD status considered at least partially recovered was within this range (57.2%).

Our study has a number of strengths. Indeed, BMD was assessed in the femoral neck, total hip, and lumbar spine by DEXA in all patients in the PRIMROSE 3 study at baseline, the end of therapy, and 24 months after the end of therapy. All DEXA scans were reviewed by a central imaging laboratory and DEXA scan quality was also centralized for each site.

Nevertheless, there are also some limitations. The number of patients in each arm was relatively small due to various circumstances like the war in Ukraine, the COVID-19 pandemic, and the inherent challenge of recruiting patients for a follow-up of 24 months. Another drawback could be the number of sites (n = 56) across different countries, but all DEXA scans were reviewed by a central imaging laboratory. Only patients from PRIMROSE 1 who were taking a placebo were randomized into two arms, placebo or linzagolix, explaining the relatively high number of black women in the placebo group. Moreover, keeping patients for such a long follow-up (24 months) in a placebo group is not easy, accounting for the small number of women in this arm.

## Conclusion

In the present study, even if the number of patients is relatively small, it can be assumed that the modest BMD changes observed from both post-treatment and pre-treatment baselines to month 24 after cessation of therapy may not have any clinically relevant effect on overall bone health of linzagolix-treated individuals, since their *Z*-scores were mostly within the expected range for age. In addition, changes in BMD values and *Z*-scores in the linzagolix treatment groups were also largely within the same range as in the placebo group, demonstrating that there are no long-term consequences on BMD after the end of linzagolix treatment.

## Data Availability

Appropriately de-identified patient-level datasets and supporting documents may be shared according to criteria established by Theramex and/or industry best practices to maintain the privacy of study participants.
